# Psychological Distress in Young Chilean Adults Exposed to Parental Alienating Behaviors during Childhood/Adolescence

**DOI:** 10.3390/ejihpe13090123

**Published:** 2023-09-03

**Authors:** Diego Portilla-Saavedra, Cristián Pinto-Cortez, Rodrigo Moya-Vergara

**Affiliations:** 1 Escuela de Psicología, Facultad de Humanidades, Universidad Católica del Norte, Antofagasta 1240000, Chile; rmoya@ucn.cl; 2 Escuela de Psicología Y Filosofía, Facultad de Ciencias Sociales y Jurídicas, Universidad de Tarapacá, Arica 1000000, Chile; cpinto@academicos.uta.cl

**Keywords:** Chile, parental alienation behaviors, psychological distress

## Abstract

The aim of this study was to analyze the psychological distress of young adults exposed to alienating behaviors during childhood/adolescence. Four hundred and sixteen adults responded to the online survey. A brief sociodemographic questionnaire, the Brief Symptom Scale, and a questionnaire on adverse childhood experiences were included. The analyses revealed that individuals who experienced one or more alienating behaviors exhibited higher levels of anxiety, depression, somatization, and overall psychological distress. Furthermore, even after controlling for the effect of other adverse childhood experiences, alienating behaviors had a significant impact on psychological distress in adulthood. This highlights an understudied aspect of alienating behaviors, i.e., their conjunction or parallelism with other adverse childhood experiences.

## 1. Introduction

Parental alienation is a relational phenomenon between a child and a rejected parent. It involves the use of aggressive behaviors by one parental figure to harm the relationship between their child and the other parental figure [1]. These aggressive behaviors have been referred to as parental alienating behaviors and could have, as their main culmination point, the child’s rejection of contact or communication with their other parent [2,3,4]. Additionally, specific parental alienating behaviors have been identified, such as denigrating the other parental figure, limiting the child’s contact with the other parent, and interfering with the communication between the child and their father or mother [2,5]. In this context, alienating behaviors have been classified as a specific form of child maltreatment (psychological/emotional) [6,7] and can manifest in different types of families (intact and separated/divorced) [8,9]. Some studies have attempted to quantify the magnitude of this phenomenon, although there are only two prevalence studies available. One of them, conducted in the United States, estimated that 13.4% of adults reported having experienced some degree of alienating behavior [10]. Additionally, another study reported that more than a quarter of adults in a sample of professionals linked to the field of social intervention reported having been exposed to alienating behaviors during their childhood [11]. This becomes significant due to the negative impact of this type of maltreatment on the mental health of individuals who have experienced it. For example, there is ample evidence of negative short- and long-term consequences for adults who have been exposed to alienating behaviors [12,13,14]. Indeed, poor mental health outcomes in adulthood have often been associated with the presence of depressive and anxious symptoms [13,15], low self-esteem, guilt, attachment issues, difficulty in other relationships, and reduced or delayed educational and professional achievement [15]. Furthermore, exposure to parental alienating behaviors during childhood has been significantly associated with similar effects as those related to psychological maltreatment, such as low self-esteem [5,16].

### The Present Study

One of the main issues associated with research on parental alienation and parental alienation behaviors is that studies have typically been conducted in North American and/or European contexts, with a predominance of developed countries [13,16]. Indeed, this has been highlighted as a gap in the evidence, as it has been suggested that the manifestations of the phenomenon itself may require cross-cultural validation, taking into account sociocultural differences among individuals, family relationships, and the countries in which the research is conducted [13]. Another relevant point to mention is that, to the extent of our knowledge, no research has been conducted on this topic in a Latin American context. In this region, researchers have generally opted for the use of other terminologies for the phenomenon, such as parental interference, family alienating practices, or the previously known parental alienation syndrome [17,18,19]. On the other hand, in various research studies that have linked parental alienation to mental health problems in adulthood, relevant aspects have been overlooked, such as the introduction of statistical control variables [12,20], including other adverse experiences of victimization from caregivers. These variables are relevant as they could contribute to understanding the shared effects that childhood experiences have on mental health in later stages of development.

Considering the previously stated facts, this study aimed to examine the relationship between parental alienating behaviors during childhood/adolescence and psychological distress symptoms in a sample of young Chilean adults. To accomplish this, two research questions were formulated:

(1) Is exposure to parental alienating behaviors during childhood/adolescence associated with psychological distress symptoms (depressive, anxious, somatic) in a sample of young Chilean adults?
(2) Does this association persist when controlling for the effect of other adverse experiences during childhood/adolescence?

## 2. Materials and Methods

### 2.1. Participants

The final sample consisted of 416 young Chilean adults, recruited through non-probabilistic convenience sampling. Participants were recruited between June and December 2022 through non-probabilistic convenience sampling. They were invited as volunteers and informed that the study focused on parent-child relationships during childhood/adolescence and adult mental health. The inclusion criteria were being between 18 to 29 years old and having both parents alive during childhood and/or adolescence. This age range was chosen because some studies have warned about the effect of recall bias in studies with retrospective designs [21,22,23]; thus, a narrower age range closer to the experienced events was considered more representative. Consequently, the final sample consisted of 416 young adults between 18 and 29 years old (M = 22.48, SD = 3.32).

### 2.2. Measures

Sociodemographic Questionnaire: A questionnaire was used to gather sociodemographic information from the participants, which they answered themselves. This instrument collected characterization information from the sample, including age, gender, educational level, and the current relationship status between parents (together/separated).

Chilean adaptation of the Baker Strategy Questionnaire—BSQ [3]: A shortened version adapted to the national context was used. This questionnaire is an 11-item measure consisting of specific behaviors in which parents might engage in order to turn the child against the other parent. Participants were asked about the presence of these behaviors during childhood or adolescence. Respondents were required to rate the same items separately for the mother (11 items) and the father (11 items) on a five-point scale, ranging from never (0) to always (4). The original version of this instrument has been used in various studies to examine the recall of exposure to parental alienation behaviors and its correlation with indicators of mental health in adulthood, showing adequate psychometric properties [5,13,16]. Some examples of items for the mother’s responses are: “She made comments that invented or exaggerated my father’s negative qualities”; while examples for the father’s responses are: “He limited or interfered with my contact with my mother, so I spent less time with her than I should or could”. In the present study, this instrument had a total α = 0.92, with α = 0.89 for responses about the mother and α = 0.91 for the father.

Short Symptom Inventory BSI-18 [24]. This is a self-report inventory that measures the frequency with which participants have experienced symptoms of somatization, depression, and anxiety in the past seven days. It consists of 18 items with 5 response options (0 = “not at all” to 4 = “very much”) and has 3 factors with 6 indicators each: somatization (items 1, 4, 7, 10, 13, and 16), depression (items 2, 5, 8, 11, 14, and 17), and anxiety (items 3, 6, 9, 12, 15, and 18). This inventory has shown adequate psychometric properties in its application in Chile as well as in other Latin American countries [25]. For the present study, this instrument had a total α = 0.93, a somatization α = 0.85, a depression α = 0.93, and an anxiety α = 0.83.

Adverse Childhood Experiences Questionnaire [26,27]: The questionnaire consists of a total of 10 questions focused on evaluating 10 different types of adversities, including aspects such as physical abuse, emotional abuse, and neglect, among others. It is responded to based on closed-ended dichotomous questions with “yes” or “no” responses for each type of adversity. This questionnaire has shown adequate psychometric properties, as evidenced by various research studies [27,28]. This questionnaire was used to introduce control variables. For the present study, only the items related to physical abuse by caregivers and neglect were utilized.

### 2.3. Procedure

Regarding the questionnaire application procedure, from June to August of 2022, the study was published on various social and professional networks (e.g., Facebook, Gmail, LinkedIn), as well as in higher education spaces. The call invited young individuals from the Antofagasta region of Chile, aged between 18 and 29 years, to participate in a research project titled “Parental Alienation in Childhood and Adolescence and its Effects on Mental Health”. The questionnaire link was distributed remotely (online) using the Google Forms platform. The entire study was designed following the principles of the Declaration of Helsinki for research involving human subjects. Additionally, it was conducted under a research protocol that was reviewed and approved by a university ethical and scientific committee. A protocol for emotional support was also developed in case participants required any psychological assistance due to their participation in the study. Lastly, it is worth mentioning that this research was reviewed and approved by the ethics committee of the University of Tarapacá, Chile, under reference number 05/22.

### 2.4. Statistical Analysis

Firstly, to examine the characteristics of the sample, descriptive univariate analyses were conducted for the main sociodemographic variables of interest. Additionally, Cronbach’s reliabilities of the considered instruments were analyzed.

In a second stage of analysis, a cutoff score was established to differentiate individuals who had experienced parental alienating behaviors during childhood/adolescence from those who had not. The dichotomous scoring of the BSQ was created by assigning a value of 0 when the participant reported no exposure to parental alienating behaviors (no Abs) and a value of 1 when any exposure to parental alienating behaviors was reported (Abs). This decision was based on criteria established in the literature and other research studies that have defined the presence and absence of parental alienating behaviors [13,20,29].

For the indicators of mental health, the BSI scales were used, and the sum of each item corresponding to each subscale was calculated, obtaining a global score for each of the three scales of psychopathological symptoms. Specifically, higher scores indicated a greater intensity of symptoms for each scale. Additionally, all items were summed to create a general psychological distress variable associated with the instrument. The skewness values were below 2 and the kurtosis values were below 7, indicating a distribution close to normal. Therefore, the variables in this study can be treated as if they were normally distributed [30]. In this regard, in order to conduct a comparative analysis, the Student’s *t*-test was chosen to compare the sociodemographic characteristics with the means of alienating behaviors first, then to compare the three symptomatology variables (somatization, depression, and anxiety) with the total scores of psychological distress between individuals who experienced parental alienating behaviors and those who did not. Additionally, Cohen’s d effect size was calculated to quantify the magnitude of the differences between the two groups.

In a third phase of analysis, to explore whether parental alienating behaviors were associated with general psychological distress, even when controlling for the effect of other types of adverse experiences in childhood/adolescence, a multiple hierarchical stepwise regression was used. This type of analysis allows us to explain the variability of the data in a dependent variable (psychological distress) based on independent variables—in this case, the alienating behaviors and the control variables. To carry this out, preliminary assumptions of the regression models were analyzed, including normality, linearity, homoscedasticity, multicollinearity, and independence. For instance, outliers and influential observations were identified using residual scatter plots. Additionally, to check the independence of residuals, which means that the errors in the measurement of the explanatory variables are independent of each other, the Durbin–Watson statistic value was examined to fall within the range of 1.5 to 2.5 [31]. For multicollinearity, two elements were considered: tolerance and the variance inflation factor. Each variable was individually entered to analyze the changes in the models. The significance level was set at *p* < 0.05. All statistical analyses were conducted using the statistical software SPSS version 25 and Rstudio.

## 3. Results

The final sample consisted of 416 young adults, of whom 59.9% identified as female, 34.4% as male, 3.1% as non-binary, and 1.1% as another gender not available in the options. Additionally, 1.9% chose not to disclose their gender. Concerning the relationship between their parents, 53.4% reported that their parents were separated/divorced, while 46.6% reported that their parents were still together. Regarding educational level, the majority of participants had incomplete university studies (56%), followed by those with completed university studies (22.2%). Detailed characteristics of the sample used in the study are presented in Table 1.

Regarding sociodemographic characteristics in relation to the quantity of reported alienation behaviors, women reported higher levels of alienation than men (t = −3105, *p* ≤ 0.001). Furthermore, there were more frequent reports of alienation behaviors in the age segment between 18 and 23 years compared to the 24–29 age group (t = 2564, *p* ≤ 0.001). There were no significant differences according to participants’ educational levels (t = 2564, *p*= 0.41). Another aspect that is significant for the literature on alienating behaviors was the relationship between parents. In this regard, as indicated by the evidence, we found significant differences among participants who reported separated/divorced parents compared to those from intact families. There was a higher prevalence of alienating behaviors reported by participants whose parents were separated (t = −5851, *p* ≤ 0.001).

Therefore, to address the established research questions, Table 2 compared the symptomatology (anxiety, depression, somatization, and general psychological distress) between individuals who reported experiencing parental alienating behaviors during childhood/adolescence (Abs, 1 or more, *n* = 339) and those who did not report them (No ABs, *n* = 77). The mean difference analysis revealed significant differences in depression symptomatology (t = 5.267, *p* ≤ 0.001), anxiety symptomatology (t = 6.436, *p* ≤ 0.001), and somatization symptomatology (t = 8.227, *p* ≤ 0.001) between individuals who reported experiencing parental alienating behaviors and those who did not. In other words, individuals who reported these behaviors displayed higher levels of depressive, anxious, and somatic symptoms compared to the group who did not report them. Furthermore, there were also significant differences in the overall score of general psychological distress (t = 7.306, *p* ≤ 0.001), indicating that individuals who reported parental alienating behaviors during childhood and adolescence experienced higher levels of general psychological distress.

Subsequently, a hierarchical multiple regression was conducted to examine the effects of parental alienating behaviors in childhood/adolescence and other adverse childhood experiences on psychological distress in adulthood. The results presented in Table 3 reveal that in Model 1, including the control variable age (Beta = −0.19, *p* ≤ 0.001), parental alienating behaviors had a significant positive effect on psychological distress (Beta = 0.40, *p* ≤ 0.001), with an R^2^ of 0.21. In Model 2, parental alienating behaviors (Beta = 0.34, *p* ≤ 0.001) maintained their significant effect, even when considering the variables of age (Beta = −0.19, *p* ≤ 0.001) and physical maltreatment by caregivers (Beta = 0.23, *p* ≤ 0.001), which also had a significant effect, resulting in an R^2^ of 0.26. For Model 3, with the inclusion of other adverse experiences as control variables, it was found that parental alienating behaviors (Beta = 0.33, *p* ≤ 0.001) maintained their significant effect, and the control variables of age (Beta = −0.21, *p* ≤ 0.001), physical maltreatment by caregivers (Beta = 0.20, *p* ≤ 0.001), and neglect (Beta = 0.16, *p* ≤ 0.001) were also significant. This model had an R^2^ of 0.28.

## 4. Discussion

The present study was conducted to increase our knowledge of the effects of exposure to parental alienating behaviors during childhood/adolescence on psychological distress in adulthood. It is important to note that this research only examined self-reported alienating behaviors and not parental alienation, which occurs when these behaviors lead to unjustified rejection of a child’s contact with one parent [32]. As a result, the phenomenon of parental alienation could not be fully examined by adhering to the perspective of Baker’s four-factor model [2] or five-factor model [32], as both highlight the necessity of the absence of any prior rights violations between the parent and the child in the alienation phenomenon.

Noting this, it is possible to infer that the association between these behaviors and mental health problems is widely documented [13,14,33]. As suggested by the literature, this study was conducted in Chile, a country that represents a different sociocultural and geographical context compared to the pioneering studies in the field, which have mainly focused on Europe and North America [11,13,20,33].

The results confirmed that individuals who reported experiencing alienating behaviors during childhood/adolescence showed higher overall psychological distress. This was assessed using an instrument like the BSI-18, which quantifies depressive and anxious symptoms, in addition to providing insight into the association of these behaviors by means of somatic symptomatology. In line with the previous literature, our findings support the idea that alienating behaviors are significantly associated with various mental health consequences in adulthood [11,13,20]. Moreover, even when the phenomenon of alienation does not manifest fully, meaning it does not culminate in the rejection of one parent by the child, experiencing some alienating behaviors alone may be sufficient to lead to worse mental health outcomes in later stages of development. This was evident in our comparisons between individuals exposed to at least one alienating behavior and those who were not. These results can be explained by the fact that alienating behaviors create an atmosphere of tension and hostility within the family dynamic, aspects that have even been compared to the impact of psychological maltreatment and family violence [1,6,7]. Another aspect that could be explanatory is that Chile is one of the countries with the highest prevalence of violence by caregivers in Latin America, particularly including psychological maltreatment and, specifically, parental interference [34]. Regarding this, it is relevant to mention that in the Hispanic literature, the concept of parental interference [19] is recognized, and is strictly linked to alienation. This is because, within the seventeen alienating behaviors described in the literature [3], acts of obstruction by a caregiver have been mentioned, which involve withholding or hiding the child or adolescent to prevent them from having contact with their other parent. This definition is similar to the one described by authors such as González Sarrió [19] and Finkelhor et al. [35] to refer to parental interference.

In a second phase of the research, our results were analyzed together with other adverse childhood experiences, such as physical abuse and neglect, that were reported by the participants. The decision to control for the effect of these variables was made because, generally, studies on alienating behaviors lack the inclusion of control variables [12,20], even though there have been attempts to do so in recent times [36]. Indeed, in addition to this, variables such as physical abuse and neglect carry significant statistical weight, and in general, in the evidence, they tend to have a greater explanatory power in terms of variance compared to other adverse experiences [37]. Taking this into account, our results indicate that even when controlling for the long-term effects of these experiences, alienating behaviors continue to have a significant effect on mental health in adulthood, as reported in various studies worldwide [12,36,38,39] which have specifically investigated alienating behaviors independently. Indeed, these results could be explained by the chronic nature of alienating behaviors over time, as individuals’ life trajectories might be marked by the presence of these circumstances [5,16], which increase their effects on mental health in a comparable way to other adverse experiences in childhood. Indeed, this opens up a new area of research on alienating behaviors that has been little explored, concerning how specific behaviors may add to other adverse childhood experiences even when full-blown alienation does not occur entirely. This nuanced perspective on alienation and its potential interactions with other adversities can provide valuable insights into the complexity of its impact on individuals’ mental health outcomes. In this sense, the evidence has demonstrated that alienating behaviors, by themselves, are already harmful to mental health [1,13,33].

However, one aspect to highlight is that, despite the evident relationship between these behaviors and psychological distress in adulthood, this association may be of low magnitude compared to other childhood adversities. This aligns with the study conducted by Baker and Verrocchio [12], which suggested that there might be protective factors for individuals who experience this phenomenon, mitigating the effects it has on their mental health. This perspective assumes an adaptive capacity of individuals facing adversities, as has been observed in other forms of childhood violence [40].

### 4.1. Implications for Practice

The findings presented herein have various associated implications. Our results can be valuable for professionals in the field of psychosocial intervention who work with families experiencing these issues, aiming to consider the impact of alienating behaviors on individuals’ mental health. Additionally, having theoretical insights based on Chilean samples could encourage authorities to view the phenomenon of alienating behaviors with greater seriousness, given the prevalent lack of information and misuse of this issue in Chile. Ultimately, the findings presented herein could serve as a foundational theoretical resource for generating campaigns aimed at preventing family violence.

### 4.2. Limitations and Directions for Future Research

However, despite following a rigorous procedure from design to analysis, it is important to acknowledge some limitations. For instance, the sample was non-probabilistic and convenience-based, so the results presented here only provide an approximation of the phenomenon of alienating behaviors in Chile. Regarding the variables of control, other adverse experiences that could involve caregiver figures were considered, making it impossible to assess the self-reporting of parental alienation per se. Furthermore, the cross-sectional nature of the research design means that causal associations cannot be assumed. It is also essential to note that the data are subject to the limitations inherent in any self-report study, such as social desirability bias. Additionally, a mention is needed of the recall bias, as it raises the possibility that participants might have omitted intricate details from their lived experiences due to memory lapses [41]. This concern is particularly pronounced in retrospective studies when measurements exhibit considerable variability and substantial temporal gaps, such as in cases of exposure to alienating behaviors during childhood or adolescence. Nevertheless, this potential issue was alleviated by taking into account the age criteria for inclusion, which involved selecting young adults. The intention behind this choice was to minimize the temporal gap between the present time and the potential recollection of exposure to alienating behaviors.

One of the main strengths of this study is that it included other adverse childhood experiences as statistical control variables, allowing for the comparison of their shared effects on adult psychological distress. Additionally, it was conducted in a developing country, Chile, breaking away from the typical studies on alienating behaviors, which are often conducted in the United States or Italy. This could contribute to the cross-cultural validity of the phenomenon. Moreover, a translated and adapted version of the BSQ was used in this study, opening up possibilities for further research using this tool.

It is essential to conduct further research that analyzes alienating behaviors in other geographical areas to determine whether this phenomenon is expressed similarly in different parts of the world. Additionally, it is necessary to have validated and culturally adapted tools for studying these behaviors in various contexts. Therefore, the development and adaptation of instruments are crucial areas of exploration for addressing alienating behaviors.

Furthermore, the consideration of other variables that could act as protective factors against these behaviors is necessary. Factors such as resilience, coping strategies, or even self-esteem, which have been mentioned in the literature, need to be empirically tested in order to understand their potential role in mitigating the effects of alienating behaviors.

## 5. Conclusions

In conclusion, the primary objective of this study was to enhance the understanding of the impact of exposure to parental alienating behaviors during childhood/adolescence on psychological distress in adulthood. The findings underscore the significance of these behaviors concerning mental health outcomes while also emphasizing the distinction between alienating behaviors and the full manifestation of parental alienation. Furthermore, this study expanded its scope beyond conventional research regions, such as Europe and North America, by investigating alienating behaviors within a Chilean context. This cross-cultural perspective provides invaluable insights into the intricate interplay between alienating behaviors and mental health within a unique sociocultural context.

## Figures and Tables

**Table 1:** Sociodemographic characteristics of the sample and differences in the means of alienating behaviors.

	*n*	%	*p*-Value
Gender			
Female	249	59.9	≤0.001
Male	143	34.4	
Age			
18–23	265	63.6	≤0.001
24–29	151	36.4	
Educational level			
Incomplete/complete high school	91	21.8	0.41
Incomplete/complete university studies	325	78.2	
Relationship status of parents			
Separated/divorced	222	53.4	≤0.001
Still together	194	46.6	

Note. The differences in sample sizes are due to response omissions. The total *n* was 416.

**Table 2:** Differences in mental health problems in adulthood in individuals reporting parental alienating behaviors in childhood/adolescence.

	ABs (1 or More)	No ABs			
Factor	M (SD)	M (SD)	t	*p*	Cohen d
Depression	10.20 (6.95)	6.16 (5.84)	5.267	≤0.001	0.62
Anxiety	9.17 (6.66)	4.76 (5.09)	6.436	≤0.001	0.74
Somatization	7.91 (6.28)	3.25 (3.96)	8.227	≤0.001	0.88
BSI-Total	27.28 (18.09)	14.1 (13.1)	7.306	≤0.001	0.83

Note: ABs (1 or more) = 1 or more reported parental alienating behaviors; No ABs = No reported parental alienating behaviors; t = Student’s *t*-test; d = Cohen’s d.

**Table 3:** Effects of parental alienating behaviors and adverse childhood experiences on psychological distress in adulthood.

Adverse Childhood Experiences	B	Desv. Error	B	t	R^2^
Model 1					
Age	−0.06	0.01	−0.19 ***	−4.46	
Parental alienating behaviors	0.02	0.00	0.40 ***	9.26	0.21
Model 2					
Age	−0.06	0.01	−0.19 ***	−4.64	
Parental alienating behaviors	0.02	0.00	0.34 ***	7.74	
Physical abuse	0.52	0.09	0.23 ***	5.36	0.26
Model 3					
Age	−0.67	0.01	−0.21 ***	−5.17	
Parental alienating behaviors	0.02	0.00	0.33 ***	7.60	
Physical abuse	0.47	0.09	0.20 ***	4.60	
Neglect	0.51	0.13	0.16 ***	3.77	0.28

Note. *** *p* ≤ 0.001. R^2^ = adjusted coefficient of determination.

## Data Availability

The data are not publicly available due to restrictions imposed by the scientific ethics committee referred to in Section 2.
